# Application of starch degrading bacteria from tobacco leaves in improving the flavor of flue-cured tobacco

**DOI:** 10.3389/fmicb.2023.1211936

**Published:** 2023-06-27

**Authors:** Yinuo Gong, Jingjing Li, Xiaohua Deng, Yiqiang Chen, Shanyi Chen, Hemin Huang, Li Ni, Teng Long, Wei He, Jianping Zhang, Zhenkun Jiang, Jianqiang Fan, Wen Zhang

**Affiliations:** ^1^Institute of Food Science and Technology, College of Biological Science and Engineering, Fuzhou University, Fuzhou, Fujian, China; ^2^Technology Center, China Tobacco Fujian Industrial Co., Ltd., Xiamen, Fujian, China

**Keywords:** microorganisms, starch degrading strains, amylase, flavor, enzyme preparation

## Abstract

Starch is an essential factor affecting the quality of flue-cured tobacco, and high starch content can affect the sensory quality and safety. Recently, the degradation of macromolecules in tobacco raw materials by using additional microorganisms to improve their intrinsic quality and safety has become a new research hotspot in the tobacco industry. However, the technical maturity and application scale are limited. Our study analyzed the correlation between microbial community composition and volatile components on the surface of tobacco leaves from 14 different grades in Fujian tobacco-producing areas. The PICRUSt software was utilized to predict the function of the microbial community present in tobacco leaves. Furthermore, dominant strains that produced amylase were screened out, and an enzyme solution was prepared to enhance the flue-cured tobacco flavor. Changes in the content of macromolecules and volatile components were determined, and sensory evaluations were conducted to assess the overall quality of the tobacco leaves. The results showed that the dominant bacterial genera on the surface of Fujian tobacco leaves were *Variovorax*, *Sphingomonas*, *Bacillus*, etc. *Bacillus* was positively correlated with various volatile components, which contributed to the sweet and aromatic flavors of Fujian flue-cured tobacco. The main genetic functions of Fujian flue-cured tobacco surface bacteria were carbohydrate metabolism and amino acid metabolism. After treating flue-cured tobacco with an enzyme preparation prepared by the fermentation of *Paenibacillus amylolyticus A17 #*, the content of starch, pectin, and cellulose in flue-cured tobacco decreased significantly compared with the control group. Meanwhile, the content of total soluble sugar and reducing sugar was significantly increased, and the volatile aroma components, such as 3-hydroxy--damascone, 2,3-dihydro-3,5-dihydroxy-6-methyl-4 H-Pyran-4-one, ethyl palmitate, ethyl linolenic acid, etc., were significantly increased. The aroma quality and quantity of flue-cured tobacco were enhanced, while impurities were reduced. The smoke characteristics were improved, with increased fineness, concentration, and moderate strength. The taste characteristics were also improved, with reduced irritation and a better aftertaste. In conclusion, *Bacillus*, as the dominant genus in the abundance of bacterial communities on tobacco surfaces in Fujian, had an essential impact on the flavor of tobacco leaves by participating in carbohydrate metabolism and finally forming the unique flavor style of flue-cured tobacco in Fujian tobacco-producing areas. *Paenibacillus amylolyticus A17 #*, a target strain with amylase-producing ability, was screened from the surface of Fujian flue-cured tobacco. The enzyme preparation, produced by the fermentation of *Paenibacillus amylolyticus A17 #*, was utilized to reduce the content of macromolecules, increase the content of water-soluble total sugar and reducing sugar, and produce a variety of crucial volatile aroma components, which had a significant improvement on the quality of tobacco leaves.

## 1. Introduction

Fujian, the birthplace of tobacco in China, has special ecological conditions suitable for growing flue-cured tobacco and is one of the critical quality tobacco areas in China. The flue-cured tobacco produced by Fujian, with its unique style, high quality, and high availability, is loved by consumers and cigarette industry enterprises. Therefore, it is essential to improve the quality of Fujian tobacco further to promote local economic development, help tobacco farmers increase their income, and eliminate poverty.

High levels of starch, pectin, cellulose, and other carbohydrate compounds in tobacco leaves can lead to an excessively burnt smell when smoked (Zong et al., 2020), produce rough smoke (Zhong et al., 2022), and increase irritation. Especially starch: the high content of starch in tobacco leaves will not only produce a burnt taste when smoked but also generate a large number of irritating gasses, such as acetaldehyde and acrolein when the starch is incompletely burned. It affects both the sensory quality and safety (Dong et al., 2015). When the content of cellulose is high, it roughens the structure of tobacco products and produces a strong choking irritation when smoked (Baliga et al., 2003; Sanders et al., 2003; Baker et al., 2005). Pectin is a hydrophilic substance that has a certain effect on the moistening properties of tobacco. However the pyrolysis of pectin produces methanol (Zhou et al., 2011), which transforms into formaldehyde and formic acid, bringing irritation and insecurity to tobacco and even affects the combustibility of tobacco, leading to an increase in tar. The protein in tobacco is a chemical component that is detrimental to the quality of flue-cured tobacco flavor, and high levels of protein increase the bitterness of the smoke during combustion and produce an odor similar to the burning of feathers (Zhou et al., 1997). Moreover, the protein in tobacco leaves is also a precursor of harmful substances such as quinoline and HCN in the smoke, which seriously affect tobacco flavor quality and safety (Slaymaker et al., 2002). In recent years, the use of additional microorganisms to degrade macromolecules in tobacco raw materials to improve the intrinsic quality and enhance the safety of tobacco has become a new research hotspot in the industry.

A large number of microorganisms were found on the surface of tobacco leaves. During the aging process, these microorganisms decompose macromolecular substances, including starch, pectin, etc., into a variety of small molecules, such as monosaccharides, amino acids, organic acids, etc., which will further react or degrade to form aroma components such as alcohols, aldehydes, acids, esters, etc., ultimately contributing to improving the smoking quality of tobacco leaves (Liu, 2011). The microbial community changes during the aging process, from sacchariferous bacteria to starch-degrading bacteria and finally to cellulosic bacteria. The change in dominant functional bacteria was linked to the gradual degradation of chemical components in tobacco leaves (Zhou et al., 2018). Various studies at home and abroad have reported using microbial fermentation to enhance the quality of low-class tobacco. For instance, surface inoculation of tobacco leaves with *Bacillus* agents and fungus agents can significantly improve the aroma quality, reduce irritation, and improve the quality of tobacco leaves (Zheng et al., 2003; Huang et al., 2021). *Bacillus* is a common functional flora in the aging process of tobacco and has been found in tobacco leaves from different regions, with α-amylase and protease activities (Chen et al., 2017). The application of an efficient amylase-producing *Bacillus thuringiensis* agent (Feng et al., 2018) to tobacco leaves resulted in a substantial reduction in starch and an increase in the content of total sugar and reducing sugar in the tobacco leaves, effectively improving the overall quality of the tobacco leaves. However, due to the stability and practicality of the microbial agents, most of the studies only remained in the laboratory for small-scale use, and it may be more feasible to utilize the enzyme preparations produced by functional microorganisms for industrial applications.

We analyzed the composition of the microbial community on the surface of flue-cured tobacco in Fujian, predicted the function of the microbial community through the PICRUSt software, and explored the potential core functional microorganisms. Based on the above research, the study further explored the different characteristics of microbial communities on the surface of different grades of tobacco leaves and analyzed the correlation between microbial communities and volatile components. In view of the high starch content in Fujian flue-cured tobacco, the *Paenibacillus amylolyticus A17 #* with safety and starch degradation ability was screened from the surface of flue-cured tobacco to be the target strain according to the sensory evaluation results. By measuring the enzyme-producing characteristics of the strain, we found that *Paenibacillus amylolyticus A17 #* could produce amylase and had the ability to produce pectinase and cellulase. Finally, the enzyme preparation was prepared for application in flue-cured tobacco, and the results of the application were evaluated by measuring the macromolecular content, volatile components, and sensory evaluation.

## 2. Materials and methods

### 2.1. Experimental materials

The 14 samples came from different tobacco growing areas in Fujian and were divided into three grades, namely high, middle, and low. Samples were provided by the Fujian China Tobacco Industry Co., Ltd. The sample information is listed in Table 1.

**Table 1:** Flue-cured tobacco samples information.

No.	Source/year/grade
F1	Fujian Sanming Youxi YLC1CB-2019 flue-cured tobacco (high-class)
F2	Fujian Longyan Yongding YLC1Y-2019 flue-cured tobacco (high-class)
F3	Fujian Longyan SanmingYLC2YCB-1-2019 flue-cured tobacco (high-class)
F4	Fujian Sanming Youxi ELC1CB-2019 flue-cured tobacco (medium-class)
F5	Fujian Longyan Yongding ELC1Y-2019 flue-cured tobacco (medium-class)
F6	Fujian Longyan Nanping SLC4-2019 flue-cured tobacco (medium-class)
F7	Fujian Longyan Sanming CF03-2019 flue-cured tobacco (low-class)
F8	Fujian Longsannan X2F03–2019 flue-cured tobacco (low-class)
F9	Fujian C-2019 stem tobacco
F10	Fujian 001–2019 thin slices
F11	Fujian Sanming Youxi SLC4F-2019 flue-cured tobacco
F12	FujianLongyan Sanming SLB3F-2019 flue-cured tobacco
F13	Fujian Sanming Youxi YLC1CB-2018 flue-cured tobacco
F14	Fujian Longyan Sanming YLC2YCB-1-2018 flue-cured tobacco

### 2.2. Strain

Test strain: *Paenibacillus amylolyticus* A17 # was screened from the surface of flue-cured tobacco samples by the Institute of Food Science and Technology of Fuzhou University, and the molecular biological identification was completed by Bioengineering (Shanghai) Co., Ltd. using the 16 S rDNA identification method with the registration number of SUB13351325 *Paenibacillus* OQ991262.

### 2.3. Main medium

#### 2.3.1. Tryptone soybean broth medium

Pancreatic Digest of Casein 17.0 g, Papaic Digest of Soybean 3.0 g, Dextrose 2.5 g, Sodium Chloride 5.0 g, Dipotassium Phosphate 2.5 g, distilled water 1,000 mL, solid medium added with 20 g (per 1,000 mL medium) of agar. pH 7.0–7.2. Autoclave at 121°C for 20 min.

#### 2.3.2. Fermentation medium

starch 1 g, bacteriological peptone 1.5 g, sodium chloride 0.06 g, potassium hydrogen phosphate 0.5 g, distilled water 100 mL, pH 7.0–7.2. Autoclave at 121°C for 20 min.

### 2.4. Microbial enrichment on the surface of flue-cured tobacco and high-throughput sequencing

In order to prepare the tobacco sample for analysis, 10 grams of aged flue-cured tobacco were added to 60 milliliters of pH 7.4 phosphate buffer in the conical flask. The mixture was shaken and washed at 200 rpm for 40 min before the washing solution was collected. A total volume of 40 mL of phosphate buffer was added to the flue-cured tobacco sample several times. The two washing solutions, which were combined after full oscillation mixing, were filtered with a 0.22-μm filter membrane to enrich the microorganisms. Ten milliliter of sterile water was utilized to wash the filter membrane. Subsequently, the bacterial solution was collected, centrifuged at 7,000 × g for 20 min, and stored at −80°C for microbial community detection. The detection and analysis of the microbial community entrusted Shanghai Meiji Bio-pharmaceutical Technology Co., Ltd. to use the Illumina MiSeq sequencing platform for high-throughput sequencing and the Meiji Bio-cloud platform for data analysis.

### 2.5. Prediction of microbial community function on flue-cured tobacco surface

PICRUSt can predict the function of metagenomes through marker gene data and a reference genome database (Langille et al., 2013). Based on 16S rRNA sequencing data, it can also be utilized to find direct evidence of the functional ability of microbial communities (Yin and Wang, 2021). The PICRUSt software calculates the abundance of different functional classes for each sample by making functional predictions based on the high-throughput sequencing results and the gene database.^1^

### 2.6. Determination of the relative content of flavor components in flue-cured tobacco

The volatile substances were extracted by headspace solid-phase extraction. The samples were crushed into powder and passed through a 425-μm sieve. After being screened, 0.4 g of the sample was put into a 20 mL sample extraction bottle, and 3 μL of phenylethyl acetate, whose concentration was 3.432 mg·L^−1^, was added as the internal standard. The headspace bottle was put into the CTC automatic sampling tray. The extraction parameter was set at 100°C for 10 min, and the volatiles were adsorbed by a 50/30 μm divinylbenzene/carboxen/poly-dimethylsiloxane (DVB/CAR/PDMS) fiber (Supelco, Bellefonte, PA, United States) for 40 min. After 4 min of analysis at 250°C at the sample inlet of the gas chromatography-mass spectrometer (model 7890B-5977A, Agilent, Palo Alto, CA, United States), the program was started.

Gas chromatography–mass spectroscopy (GC–MS) conditions: a Hp-5 ms chromatographic column (60 m × 0.25 mm × 0.25 μm) was used. The carrier gas is 99.999% pure helium at a flow rate of 1 mL· min^−1^. The temperature program was as follows: the initial temperature was held at 50°C for 2 min and programmed to increase at 8°C·min^−1^ to 280°C, and then was held for 25 min. The post-run temperature was 280°C, and the post-run time was 5 min. The Splitless injection was used. The solvent delay was 4 min.

Mass spectrum conditions: the electron energy was 70 eV, and the ion source temperature was 230°C. The interface temperature was 280°C, and the transfer line temperature was 150°C. The scanning mode was full scanning and the mass scanning range was 35–350 m/z.

Qualitative and quantitative analysis of volatile substances: The total ion chromatogram of volatile substances were obtained by GC–MS analysis and were retrieved by the NIST 14 spectrum library. Qualitative analysis was carried out together with reference documents, and semi-quantitative calculation was carried out for each component based on the concentration of the internal standard. Content of volatile components (μg·L^−1^) = concentration of internal standard × three × Peak area of component/peak area of internal standard.

### 2.7. Screening of starch-degrading strains on the surface of flue-cured tobacco

In the early stage of the experiment, a batch of strains producing starch hydrolysis circles in the amylase screening medium plate were isolated from the surface of Fujian flue-cured tobacco. The strains with a large hydrolysis circle were selected for further comparison of amylase activity in the fermentation broth; incidentally, the fermentation broth was evenly sprayed on the cut tobacco for sensory evaluation. Finally, *Paenibacillus amylolyticus A17 #*, with strong amylase activity that has significantly improved the sensory of flue-cured tobacco, was selected for follow-up tests.

### 2.8. Fermentation of the strain and enzyme preparation

The activated *A17 #* strain was incubated in TSB medium at 200 r·min^−1^ and 37°C for 12 h at a constant temperature, and 5 mL (per 100 mL fermentation liquor) of bacterial liquid was taken into the fermentation medium. The fermentation conditions were 37°C and 200 r·min^−1^ for 24 h. Based on the fermentation medium, the fermentation broth was taken at 3 h, 6 h, 12 h, 18 h, 24 h, and 36 h to determine the growth of the strain (calculated by OD_600nm_). The fermentation broth was centrifuged at 9,000 × g at 4°C for 10 min, and its supernate (crude enzyme solution; Chen et al., 2017) was concentrated and filtered through ultrafiltration membranes of 100 kDa and 30 kDa to produce the liquid amylase preparation for enzymatic activity determination.

The determination method of amylase activity is adjusted with reference to Yoo’s modified method (Yoo et al., 1987). The amylase activity is defined under the conditions of pH 5.8 and temperature 37°C, and the amount of enzyme required to hydrolyze 1 mg of starch in 5 min is one enzyme activity unit (U), expressed in U·mL^−1^.

The determination method of pectinase activity refers to the determination method of pectinase activity of Tang et al. (2019). The pectinase activity is defined under the conditions of pH 9.4 and temperature 50°C, and the amount of enzyme required to catalyze pectin hydrolysis to form 1 μg of galacturonic acid is one enzyme activity unit (U), expressed in U·mL^−1^.

The determination method of cellulase activity refers to the determination method of cellulase activity of Bedade et al. (2017). The cellulase activity is defined under the conditions of pH 5 and temperature 50°C, and the amount of enzyme required for the hydrolysis of CMC-Na to form 1 μg of glucose in 1 min is one enzyme activity unit (U), expressed in U·mL^−1^.

The determination method of protease activity is determined according to the national standard GB23527-2009 Folin method. The protease activity is defined under the conditions of pH 7.5 and a temperature of 40°C, and the amount of enzyme required for the hydrolysis of casein to form 1 μg of tyrosine is one enzyme activity unit (U), expressed in U·mL^−1^.

### 2.9. Determination of macromolecular substances

The contents of pectin and cellulose were determined with reference to YC/T 346–2010 (Tobacco and tobacco products—Determination of pectin, 2010) and YC/T 347–2010 (Tobacco and tobacco products—Determination of neutral detergent fiber, acid detergent fiber and acid detergent lignin, 2010) respectively. Starch and protein were determined by using an automatic continuous flow analyzer (France Aliance, model Futura) according to standards YC/T 216–2013 (Tobacco and tobacco products—Determination of starch, 2013) and YC/T 249–2008 (Tobacco and tobacco products—Determination of protein, 2008) respectively.

### 2.10. Determination of conventional chemical composition

Total alkaloids, water-soluble total sugar, and reducing sugar were determined by the continuous flow method, using the same macromolecular content determination equipment as above, and referring to YC/T 160–2002 (Tobacco and tobacco products—Determination of total alkaloids, 2002), YC/T 217–2007 (Tobacco and tobacco products—Determination of Potassium, 2007) and YC/T 159–2019 (Tobacco and tobacco products─Determination of water soluble sugars, 2019) respectively.

### 2.11. Sensory evaluation test

The redried tobacco leaves that had been made into cut tobacco from the lower grade SLC4F-2020 in Nanping, Fujian Province, were selected as the samples for the sensory test. Simulate the workshop operation process: 50 g of tobacco leaves were taken and laid on the sample tray, and the amylase solution was evenly sprayed on the tobacco leaves by a throat sprayer at an application rate of 1,000 U·kg^−1^ of tobacco at a 5% application ratio. Subsequently, the treated tobacco leaves were put in a bag at room temperature for 6 h for enzymatic hydrolysis. Later, the enzyme was inactivated at 135°C for 1 min. The treated samples were bagged in a constant temperature and humidity incubator at 22°C and (60 ± 5) % relative humidity to balance the moisture for 48 h. In the meantime, the same amount of water and inactivated enzyme solution were taken as the control. Finally, the samples obtained were made into cigarettes, randomly numbered, and then distributed to 10 professional reviewers for sensory evaluation. Refer to GB 5606.4–2005 (Cigarettes, 2005). The six items of each cigarette, including aroma, delicacy, impurity, irritation, etc., were evaluated and scored, and the results of the sensory evaluation were presented as the average scores of 10 experts.

### 2.12. Statistical analysis

All samples were conducted at least in triplicate and the results were expressed as means ± standard deviation. Statistical analysis of the data was performed using Statistical Package for the Social Sciences (SPSS) 23.0 (IBM, Armonk, New York). Student’s t-test was used for two-group comparisons (*p < 0.05, **p < 0.01),

## 3. Results

### 3.1. Correlation between microbial diversity and flavor of flue-cured tobacco surface

#### 3.1.1. The microbial diversity of flue-cured tobacco surface

**Figure 1:**
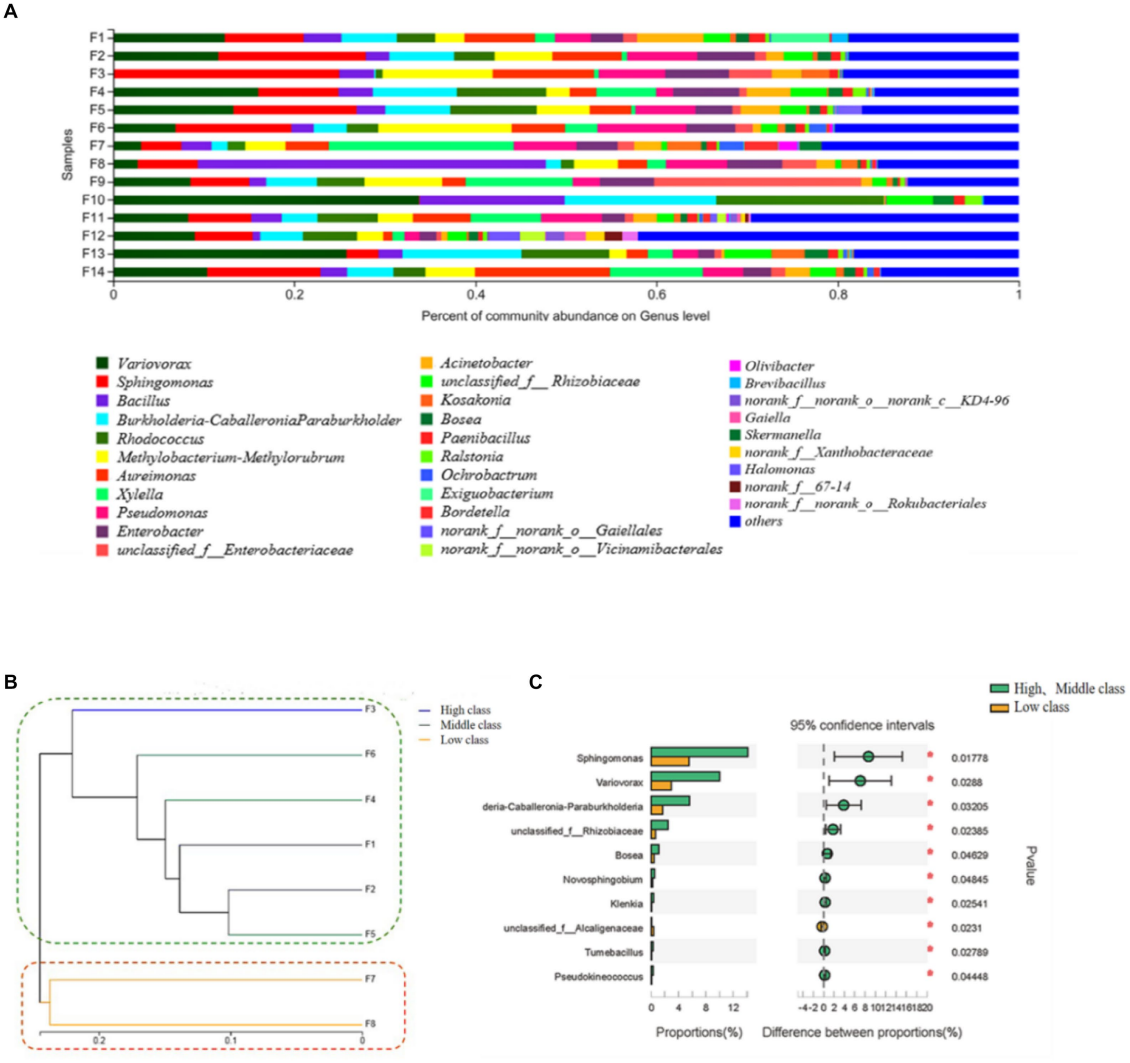
Relative abundance stacking diagram **(A)**, cluster analysis tree graph **(B)** and difference analysis **(C)** of bacterial communities on the surface of flue-cured tobacco samples with different quality grades. The serial numbers correspond to different grade of flue-cured tobacco samples from Fujian tobacco-producing areas. The vertical coordinates were the name of the samples, the horizontal coordinates were the proportion of the genera in the samples, the columns of different colors represent different genera, and the length of the columns represents the proportion of the genera. High-class: F1; F2; F3; Middle-class: F4; F5; F6; Low-class: F7; F8.

At the genus level, the top 10 dominant bacterial species in the bacterial community abundance on the surface of Fujian tobacco leaves were *Variovorax*, *Sphingomonas*, *Bacillus*, *Burkholderia-Caballeronia-Paraburkholderia*, *Rhodococcus*, *Methylbacillus*, *Aureomonas*, *Pseudomonas*, *Bacillus* and *Ralstonia* (Figure 1A). It had been reported that *Pseudomonas* could degrade the content of macromolecular compounds and increase the content of ionone and fumarone, which could improve the rose fragrance in tobacco leaves (Yu et al., 2021). *Bacillus* could promote the fermentation of tobacco and the formation of flavor compounds (English et al., 1967). During the fermentation process of leaves such as *Pu′er* tea, it was found that *Bacillus*, as the main metabolic bacteria (Xu et al., 2019), could produce pectinase, amylase, and another hydrolase to increase the content of soluble sugar and ensure the power for after fungal fermentation. In addition, *Variovorax,* which had the highest percentage of the bacterial community on the surface of Fujian tobacco leaves (11.56%), was the main microorganism in the plant rhizosphere. *Variovorax* was often found in the soil environment and plant growth process (Gao et al., 2020), which had the function of promoting plant growth and development. *Bacillus amyloliquefaciens,* with the characteristic of secreting complex enzymes (protease, amylase, cellulase, pectinase, and peroxidase), could improve the aroma quality and quantity of tobacco leaves during the aging process (Tobacco and tobacco products—Determination of Potassium, 2007; Tobacco and tobacco products─Determination of water soluble sugars, 2019). These dominant bacteria played different roles in the diversity of the bacterial community on the surface of Fujian tobacco. Finally, they formed the unique flavor style of flue-cured tobacco in Fujian tobacco-producing areas.

Further analysis of the microbial community in different grades of tobacco samples obtained the cluster analysis tree graph **1 (B)** of tobacco samples. The tobacco leaves samples were divided into two categories by the bacterial community. The high-class tobacco (F1, F2, F3) and the middle-class tobacco (F4, F5, F6) intersected with each other and were separated from the low-class tobacco (F7, F8) samples (Figure 1B), indicating that the composition of the microbial community was related to the quality of tobacco leaves. The difference in bacteria between the high, middle, and low-class flue-cured tobacco was analyzed by the Welch *T*-test. From the perspective of the bacterial community, most of the different bacterial genera were up-regulated in the high- and middle-class tobacco, and *Sphingomonas* and *Variovorax* were the main species differences in Fujian tobacco leaf samples (Figure 1C). Previous studies had shown that *Sphingomonas* could tolerate extremely poor nutritional conditions (Huang et al., 2009), making it advantageous in degrading aromatic substances, and it could produce valuable macromolecular polymers such as carotenoids and other odorogenic precursors (Kikukawa et al., 2021). Meanwhile, *Variovorax* not only played a role in plant growth (Gao et al., 2020) and disease resistance, but it also degraded isoprene compounds (Dawson et al., 2020), and usually, the derivatives degraded by isoprene may form aromatic substances.

#### 3.1.2. Analysis of volatile aroma components of different grades of flue-cured tobacco

**Figure 2:**
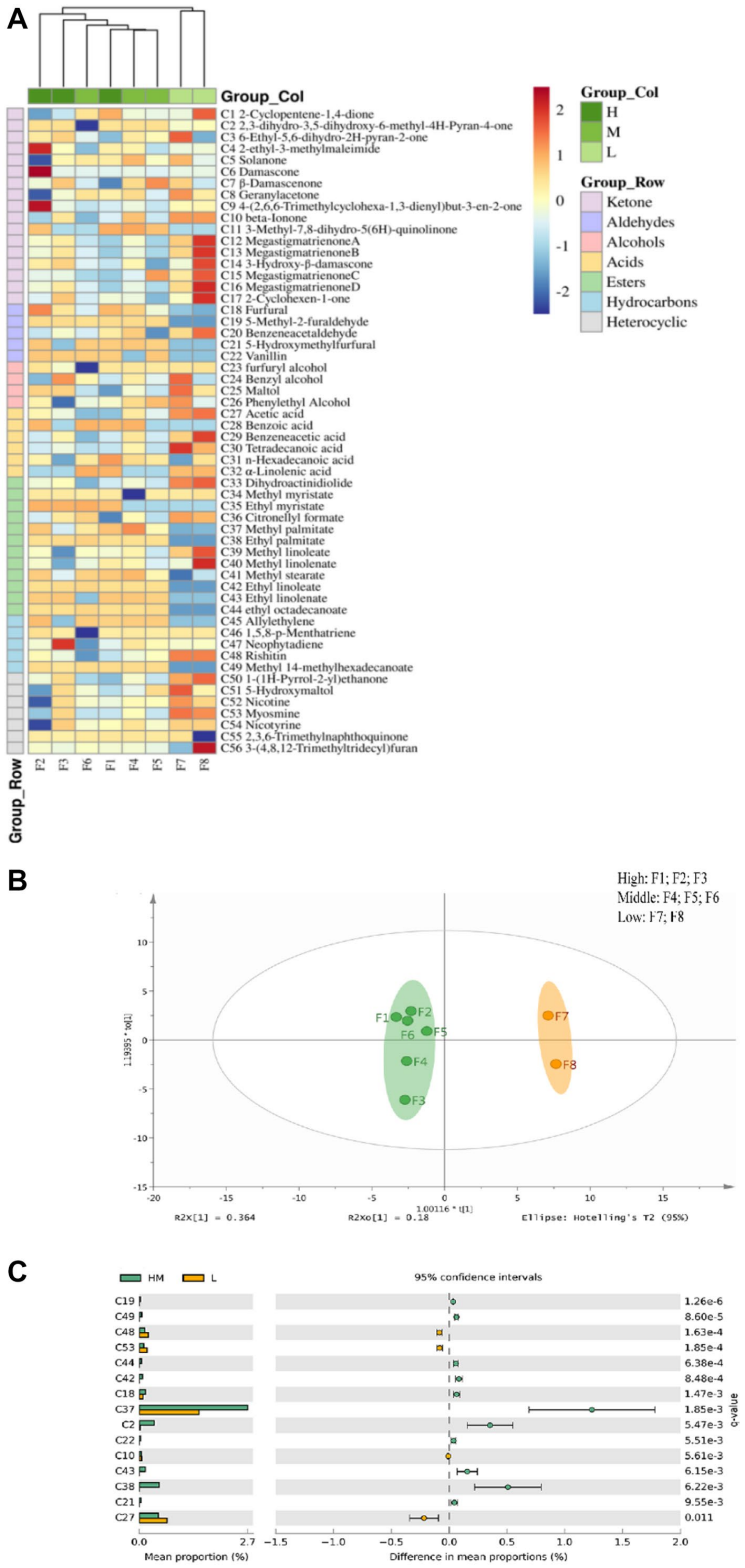
Correlation heat map **(A)** and OPLS-DA analysis chart **(B)** and differential volatile component histogram **(C)** of volatile aroma components in flue-cured tobacco samples of different grades.

The volatile aroma components of flue-cured tobacco have a decisive impact on the quality (Liu et al., 2022). Through SPME-GC–MS technology, the volatile aroma components of different grades of tobacco were detected, and the cluster heat map was drawn. A total of 56 volatile aroma components were obtained, including 18 ketones, 12 esters, 7 heterocycles, 7 hydrocarbons, 6 acids, 5 aldehydes, and 4 alcohols. Ketones were the main components that form the delicate aroma of tobacco leaves (Figure 2A). Esters had a certain impact on the aroma and taste of tobacco leaves, which could increase the pure and mild sense of smoke (Zuo et al., 2020). It can be seen from the hierarchical clustering of tobacco samples that tobacco samples of different grades were divided into two categories, while the volatile substances of high and middle-class tobacco were not significantly different from each other and were grouped into one category, but they were distinguished from the low-class tobacco.

Further, the volatile aroma components in the high, middle, and low-class tobacco samples were analyzed by the OPLS-DA model, and the differences in the volatile aroma components in the two samples were analyzed by Welch’s t-test. The volatile substances in the high and middle-class flue-cured tobacco were similar, which were quite different from those in the low-class tobacco samples (Figure 2B). According to Figure 2C of the difference analysis, the relative content of volatile aroma components in high- and middle-class tobacco was higher (*p* < 0.05). Among them, 5-methyl furfural (C19) had a sweet and caramel flavors. Ethyl stearate (C44) had a waxy flavor. Furfural (C18) had fruit and caramel flavors. 5-hydroxymethylfurfural (C21) had flower and caramel flavor. Vanillin (C22) had vanilla flavor and sweet milk flavor (Mao et al., 2020) that could highlight the characteristics of medium and high-class tobacco leaves with a sweet smell and weak flower fragrance. The content of sweet mixed esters such as ethyl palmitate (C38) increased, which improved the plumpness and mellowness of the smoke of medium- and high-class tobacco leaves. In addition, the content of nicotine components such as methamine (C53), leading to strong irritation in low-class tobacco, was high.

#### 3.1.3. Correlation analysis of microbial species and volatile components on the surface of flue-cured tobacco

**Figure 3:**
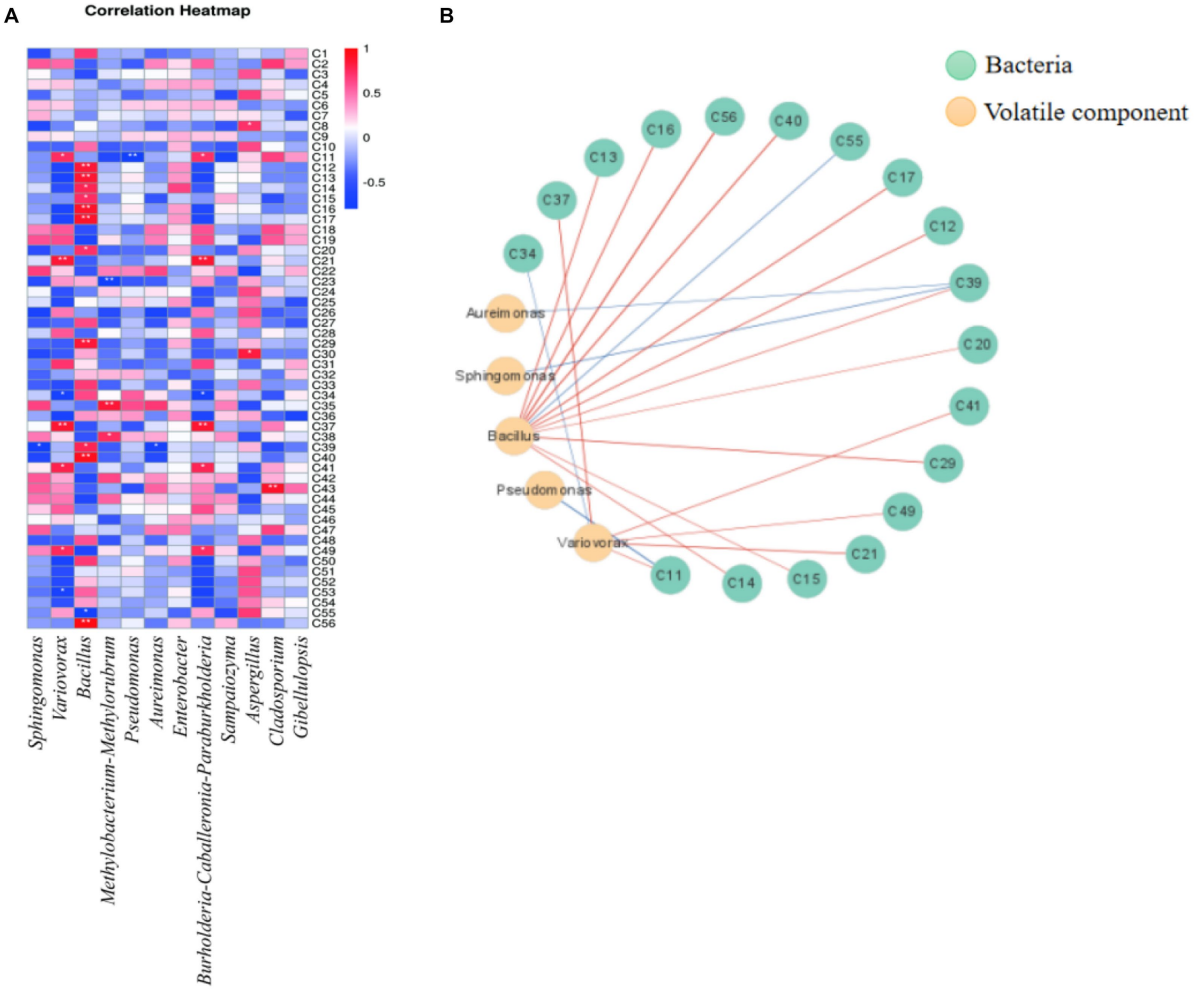
Correlation heat map and correlation network analysis of microbial community and volatile components **(A)**: correlation heat map; **(B)**: Correlation network diagram. (red line indicates positive correlation, blue line indicates negative correlation).

The above analysis found that the clustering results of microbial communities in flue-cured tobacco of different grades were similar to those based on volatile aroma components. In other words, the flue-cured tobacco samples could be divided into two (high/middle and low) categories according to the clustering of microbial diversity and volatile aroma components. For this reason, this study further analyzed the relationship between the microbial community on the surface of different grades of flue-cured tobacco and the volatile aroma components. It can be seen that bacterial genera such as *Bacillus* and *Variovorax* had an important impact on the volatile aroma components in tobacco leaves (Figure 3A).

The correlation network diagram 3 (B) was drawn with Cytascape software. As shown in Figure 3B, 21 paired correlations were obtained (| r | > 0.6, *p* < 0.05), among which 18 volatile aroma components were related to bacteria. Bacillus has a large number of related connections, which was positively correlated with megastigma-trienone A (C12), megastigma-trienone B (C13), megastigma-trienone C (C15), megastigma-trienone D (C16), etc. Megastigma-trienones were important components that contributed to the aroma of flue-cured tobacco (Chida et al., 2004). Among them, Megastigma-trienone C had a unique dry grass-like sweetness that could significantly enhance the sweetness of tobacco and create a smoother smoke (Zhan et al., 2011). It was inferred that *Bacillus* strains had a significant impact on the formation of flavor in flue-cured tobacco. Furthermore, the presence of *Variovorax* bacteria had been found to be positively correlated with the presence of neophytadiene (C41), an important aroma component in flue-cured tobacco that could enhance its fragrance (Zhou et al., 2005).

#### 3.1.4. Function annotation of KEGG database of bacterial community

**Figure 4:**
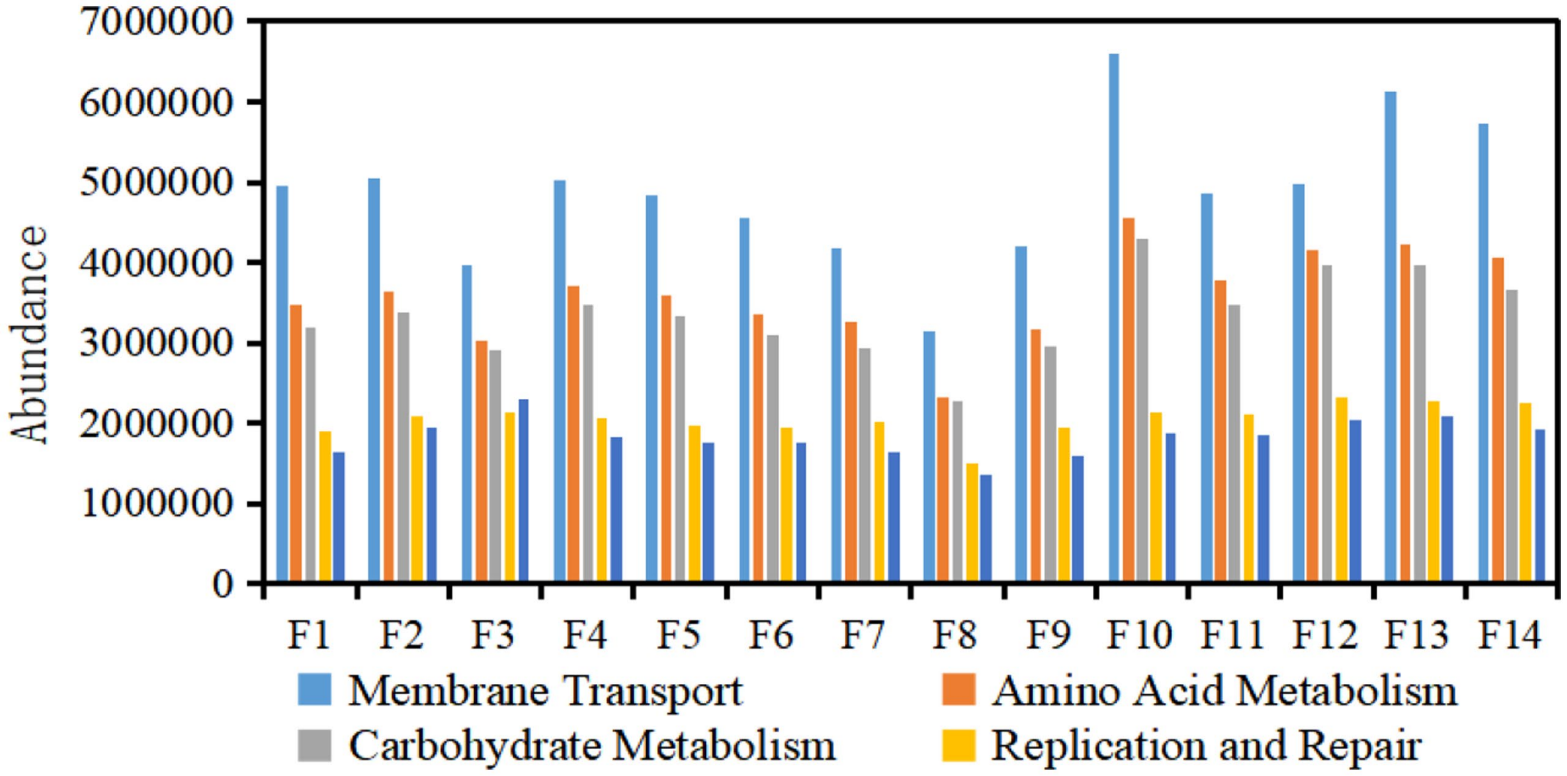
Abundance of KEGG metabolic pathway of bacterial community in flue-cured tobacco samples.

The high-throughput sequencing results of samples show that there were enzyme systems. Based on the functional metabolic pathway of the second layer of the KEGG database, the functional abundances of the metabolic pathway of the bacterial community in the top 5 in all samples of each tobacco sample were plotted in column chart 4. The main genetic functions of Fujian flue-cured tobacco surface bacteria were carbohydrate metabolism and amino acid metabolism (Figure 4). On the one hand, the intermediate products of carbohydrate metabolism could promote reactions such as the synthesis of aromatic amino acids (Xu et al., 2018) and provide aroma precursors. On the other hand, reducing sugars could react with amino acids in an important Maillard reaction to produce a class of important aroma substances in flue-cured tobacco. These two metabolic pathways were closely related to the quality of tobacco leaves, and bacteria were fully involved in the metabolism of amino acids and carbohydrates, which were important components of the microecology in the aging process of tobacco leaves. It was speculated that the bacterial community on the surface of Fujian flue-cured tobacco may have an important impact on the flavor of tobacco leaves by participating in carbohydrate metabolism.

### 3.2. Screening of starch-degrading strains on the surface of flue-cured tobacco

A total of 80 strains were isolated and purified from the surface of 14 Fujian flue-cured tobacco samples, of which 7 strains had strong starch-degrading abilities in a starch-solid screening medium. Based on the results of the smoking evaluation after spraying the crude enzyme solution of the strain on the flue-cured tobacco, strain *A17 #,* identified as *Paenibacillus amylolyticus,* was screened, which could significantly improve the quality of tobacco leaves.

**Figure 5:**
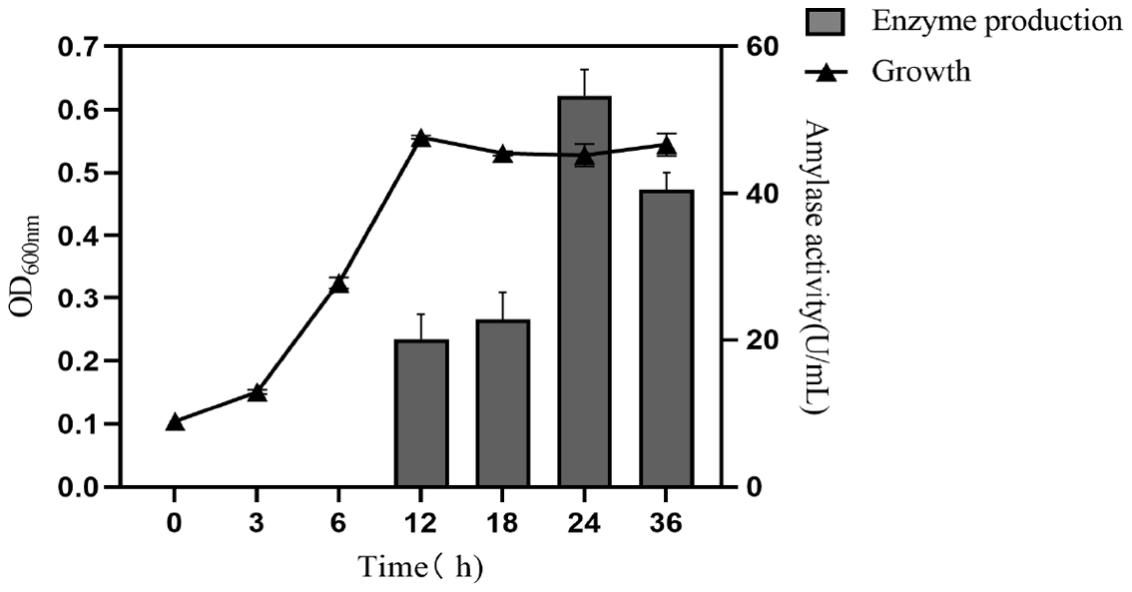
The growth and enzyme-producing curve of *Paenibacillus amylolyticus A17 #* strain in the fermentation medium.

According to the determination of its enzyme-producing growth curve, which was shown in Figure 5. *Paenibacillus amyloliquefaciens A17 #* grew logarithmically in 0–12 h under the basic enzyme-producing medium and was in a stable phase after 12 h. Amylase was produced at 12 h and rapidly accumulated at 18–24 h. To sum up, the strain reached the peak of enzyme production at the stable growth stage, and the highest amylase activity was 53.28 U·mL^−1^ at 24 h. With the prolongation of fermentation time, amylase activity showed a downward trend in 24–36 h due to the reduction of nutrients, the accumulation of waste, the inhibition of cell death, and the decomposition of metabolites (Mao et al., 2020).

**Figure 6:**
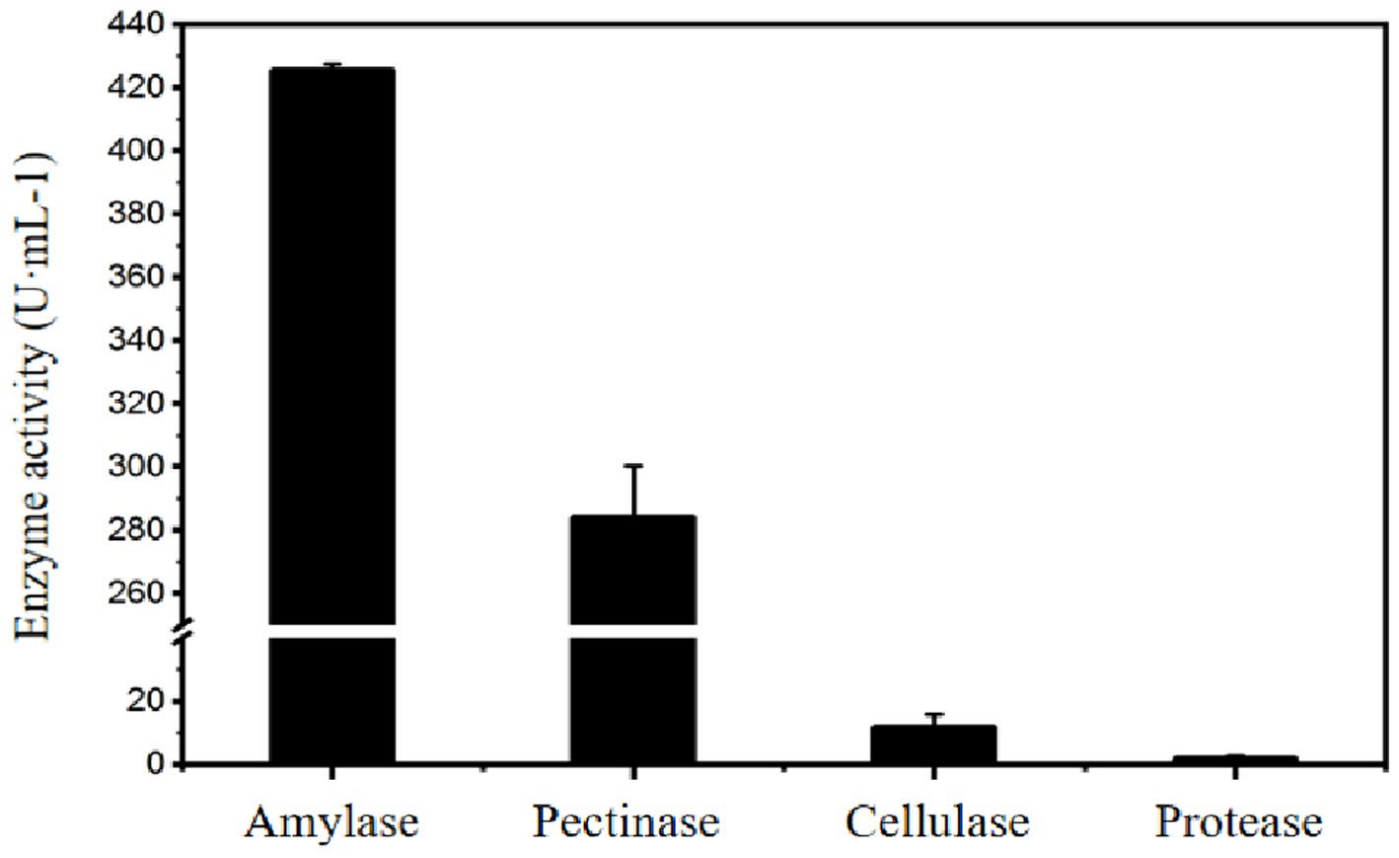
Enzymatic characteristics of *Paenibacillus amylolyticus A17 #* strain.

Meanwhile, the cellulase, pectinase, and protease activities of the liquid enzyme preparation of *Paenibacillus amyloliquefaciens A17 #* strain were measured. The results show that the *A17 #* strain could produce pectinase and cellulase in addition to producing more amylase under the starch-induced enzyme-producing medium, and the pectinase activity can reach 280 U·mL^−1^ (Figure 6), which indicated that the preparation of *Paenibacillus amyloliquefaciens A17 #* was a complex enzyme system.

### 3.3. Changes in the content of macromolecular substances in flue-cured tobacco after the enzyme treatment

**Figure 7:**
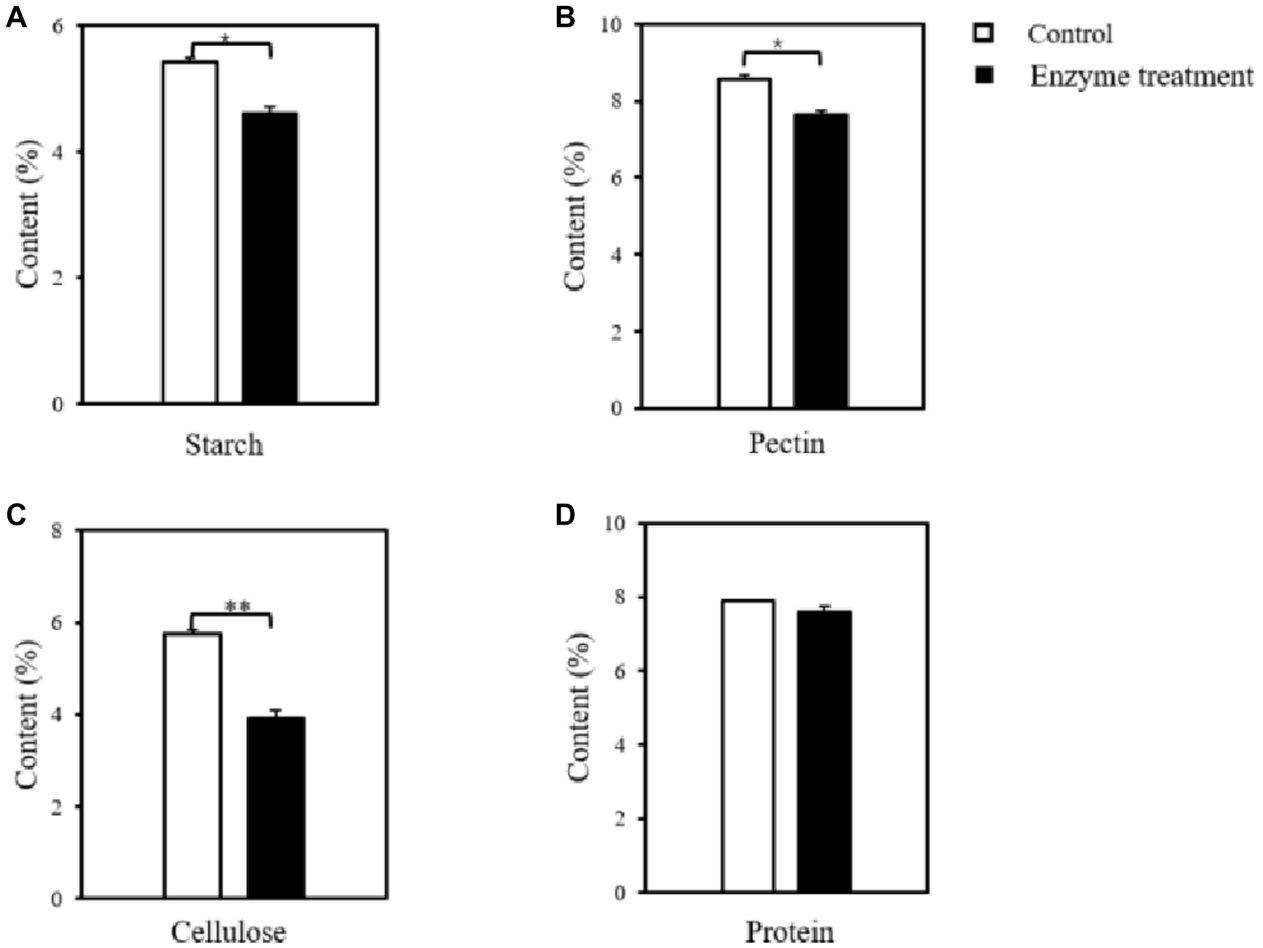
Changes in the content of macromolecular substances in flue-cured tobacco before and after the enzyme treatment. **(A)** starch; **(B)** pectin; **(C)** cellulose; **(D)** protein; ^*^p < 0.05, ^**^p < 0.01.

The content of macromolecular substances in tobacco leaves before and after the treatment of enzyme preparation was determined on the basis of taking the inactivated enzyme-treated group as the control group. As shown in Figure 7A, the starch content in the flue-cured tobacco was significantly reduced after the enzyme treatment, with a decrease of 15.05% relative to the control, indicating that the enzyme treatment achieved the degradation of starch in the flue-cured tobacco. Moreover, after measuring the content of other macromolecules, it was found that the content of pectin (Figure 7B) and cellulose (Figure 7C) were significantly reduced, decreasing by 10.71 and 31.89%, respectively, compared with the control group. While the content of protein (Figure 7D) did not change significantly after the enzyme treatment. These results further revealed the microbial enzyme preparation was a compound enzyme that can degrade the cell wall substances of tobacco leaves. It was found that pectinase could cleave the intercellular layer and primary wall of tobacco leaves α- 1,4 glycosidic bond, which loosens the binding between the concentric layers of fibers, and further plays the role of cellulase to break the cellulose structure of the cell wall (Wang et al., 2020). Combined with the results of enzymatic activity characterization of enzyme preparation in the early stage, it is speculated that the synergism of gelatinase and cellulase causes a large decrease in cellulose content. Based on the change in macromolecule content and the subsequent sensory evaluation results, it was confirmed that reducing the macromolecule content in tobacco leaves could significantly improve the original defects of tobacco leaves, such as scorching and choking, and improve the quality of tobacco leaves.

### 3.4. Changes in the content of main chemical components in flue-cured tobacco after the enzyme treatment

**Figure 8:**
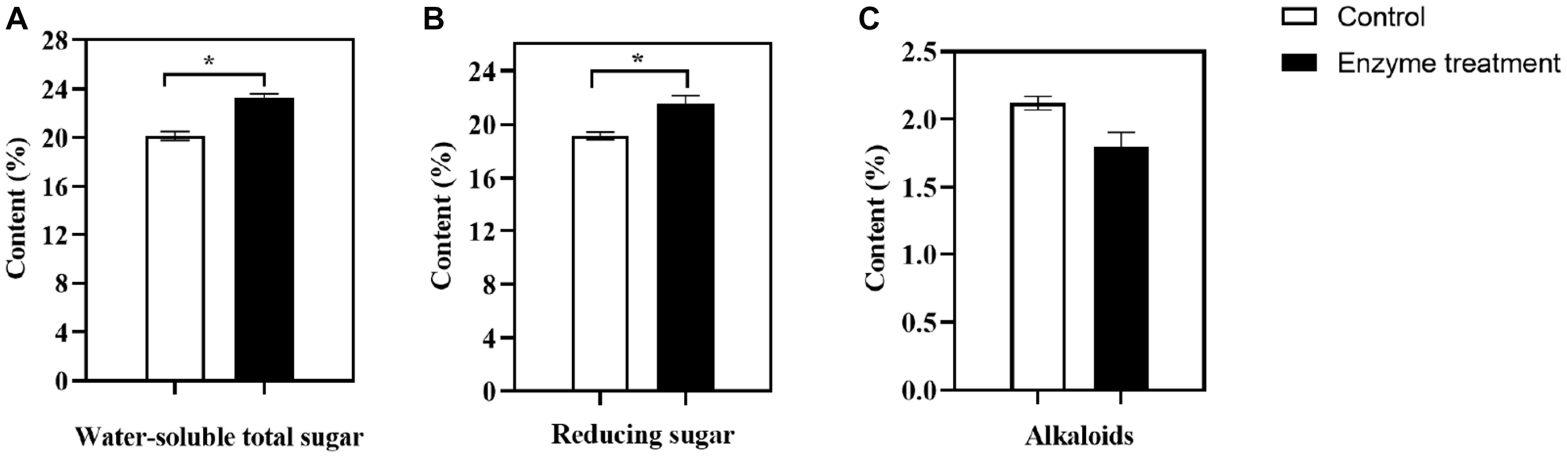
Changes of main chemical components in flue-cured tobacco before and after the enzyme treatment. **(A)** water-soluble total sugar; **(B)** reducing sugar; **(C)** alkaloids; ^*^p < 0.05, ^**^p < 0.01.

The content of water-soluble total sugar and reducing sugar in tobacco leaves are important parameters in the routine chemical analysis of flue-cured tobacco. These parameters are critical in determining the mellowness of flue-cured tobacco smoke and serve as key indicators of its overall quality (Fan, 2017). In our study, the content of total water-soluble sugar (Figure 8A) in the flue-cured tobacco was significantly higher in the enzyme-treated group, with an increase of 3.13%, and the content of reducing sugar (Figure 8B) was significantly increased by 2.40% compared to the control group. The enzyme-treated group had been found to significantly increase the content of water-soluble sugar and reducing sugar, which was an important factor in the enhanced sweetness of the flue-cured tobacco and a more delicate and balanced smoke.

The level of sugar in tobacco products is an important factor in determining the preferences of both tobacco producers and consumers. Typically, tobacco products with sugar content ranging from 8 to 30% are preferred (Talhout et al., 2006). Alkaloids, which mainly consist of more than 90% nicotine (Zhou et al., 2012), are responsible for the strength of smoke. However, when the content of alkaloids is too high, it can cause discomfort, such as choking and irritation, and even pose risks to human health. In this study, it was found that the content of alkaloids (Figure 8C) in flue-cured tobacco after the enzyme treatment showed a downward trend, with a decline of 0.32%, which could further enhance the safety of tobacco.

### 3.5. Changes of volatile components in flue-cured tobacco after the enzyme treatment

The inactivated enzyme-treated group was used as the control group. The volatile components in flue-cured tobacco were analyzed using HS-SPME/GC–MS technology. A total of 68 volatile substances were identified, including 15 ketones, 6 aldehydes, 3 alcohols, 6 acids, 19 esters, 10 heterocyclic compounds, and 9 other compounds.

**Figure 9:**
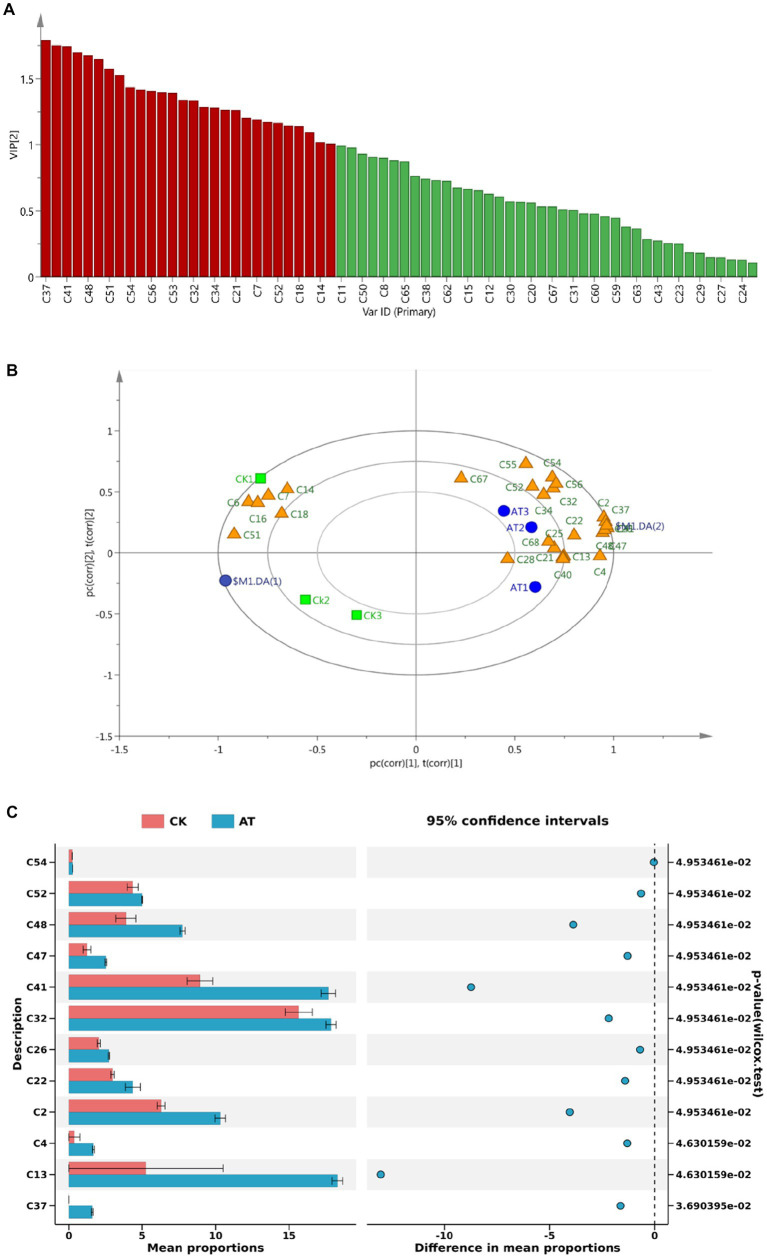
VIP value **(A)** and load diagram **(B)** of volatile substances in flue-cured tobacco before and after the enzyme treatment and significance analysis of main volatile substances **(C)**. Enzyme-treated group: AT1, AT2, AT3; Inactive enzyme-treated group: CK1, CK2, CK3. The data of volatile substances in flue-cured tobacco before and after the enzyme treatment was supplied in Supplementary Table 1.

The value of variable importance in the projection (VIP) can be used to quantify the contribution of each variable to the classification (Liu et al., 2018). In the study, the VIP values of volatile components in flue-cured tobacco before and after the enzyme treatment were calculated using SIMCA software (v 14.1, MKS Umetrics AB). A total of 29 volatile components were found to have VIP values greater than 1 (Figure 9A), indicating their significant contribution to the classification. The load diagram showed that there were more volatile aroma components near the position of the enzyme-treated group (AT), indicating that the aroma components of flue-cured tobacco after the enzyme-treated were richer and the aroma characteristics were more obvious (Figure 9B).

The significance analysis of the important volatile components (VIP > 1) screened out is shown in Figure 9C. Compared with the control group, 2,3-dihydro-3,5-dihydroxy-6-methyl-4H-Pyran-4-one (C2), phenylacetic acid (C26), ethyl 13-methyl tetra decanote (C37), ethyl palmitate (C41), ethyl linoleate (C47), and linolenic acid ethyl ester (C48) in the enzyme-treated group were significantly increased. Phenylacetic acid (C26), with a sweet and floral fragrance, and ethyl palmitate (C41) which causes the sweet sense were the products of the Maillard reaction (Li et al., 2017). Previous studies have shown that the significant increase in the content of Maillard reaction products not only has a positive and direct contribution to the improvement of the aroma quality, aroma quantity, aftertaste, and total smoking score of tobacco leaves but also has an indirect effect on the reduction of impurities and irritation (Shi et al., 2013; Wang et al., 2019). After the enzyme treatment, sweet esters such as ethyl linoleate (C47) and ethyl linolenic acid (C48) increased significantly. The ester components have a certain impact on the aroma and taste of tobacco leaves, which could increase the mellow feeling of flue gas (Zuo et al., 2020).

### 3.6. Sensory evaluation results of flue-cured tobacco after the enzyme treatment

**Figure 10:**
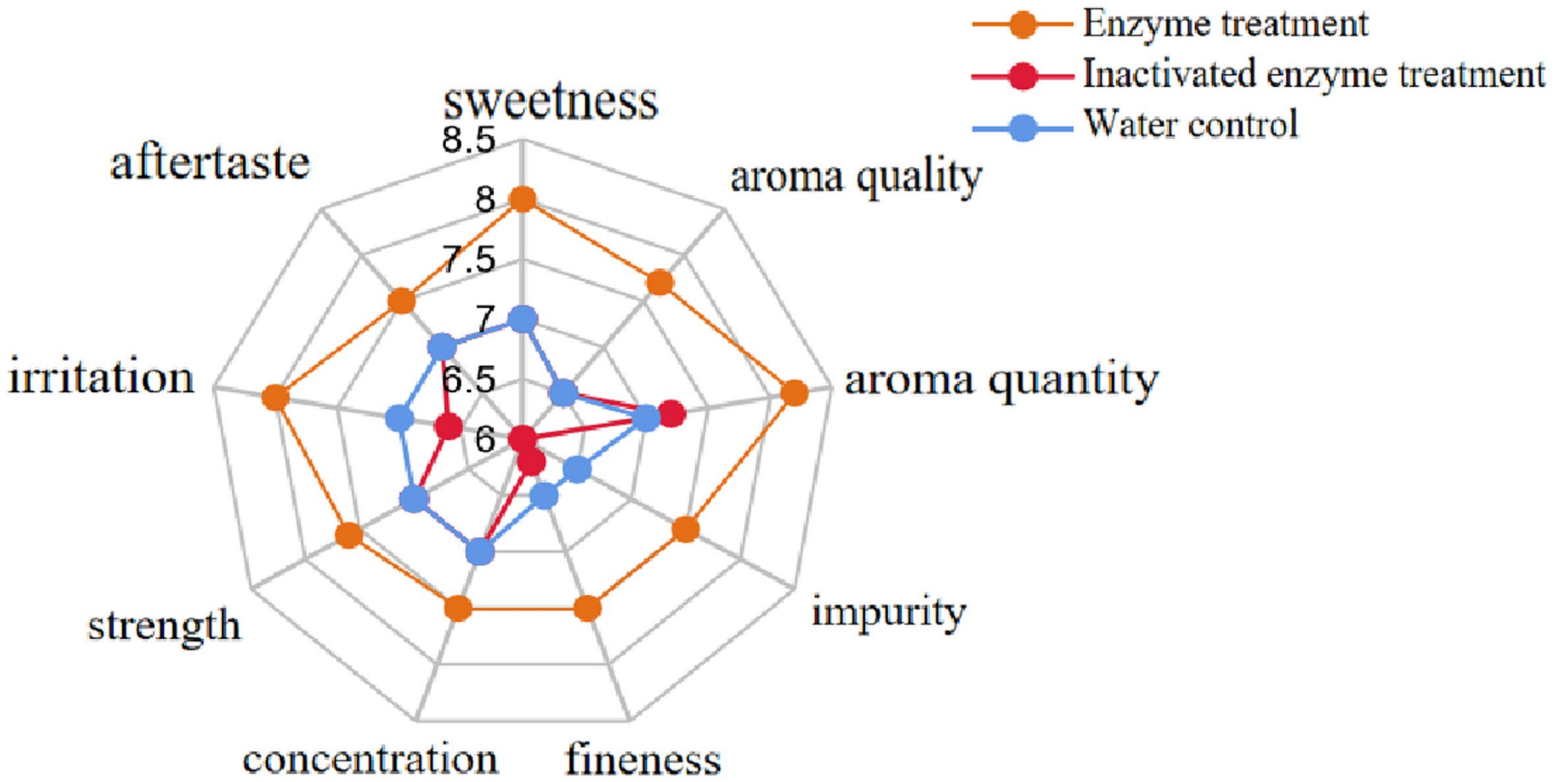
Radar chart of smoking evaluation scores of different treatment groups.

The enzyme preparation was evenly sprayed on the tobacco leaves by a throat sprayer at an application rate of 1,000 U·kg^−1^ of tobacco at a 5% application ratio, while two control groups were sprayed with the same amount of water and inactivated enzyme solution, respectively. The ten professionals evaluated treated tobacco leaves based on four aspects: style characteristics (sweetness), aroma characteristics (aroma quality, aroma quantity, impurity), smoke characteristics (fineness, concentration, strength), and taste characteristics (irritation, aftertaste). The results showed that the enzyme treatment improved the style characteristics of the tobacco leaves, making them sweeter. The aroma quality and quantity were also enhanced, while impurities were reduced. The smoke characteristics were improved, with increased fineness, concentration, and moderate strength. The taste characteristics were also improved, with reduced irritation and a better aftertaste (Figure 10). In conclusion, enzymatic hydrolysis could effectively improve the characteristics of flue-cured tobacco, making it sweeter, with better aroma, smoke, and taste characteristics.

## 4. Discussion

Microorganisms play a vital role in the aging process of tobacco leaves. Previous studies show that they shorten the aging cycle and can improve the taste and flavors of tobacco products (Huang et al., 2010). Edgar (2013) and Edgar et al. (2011) suggested that *Bacillus* species played an essential role in enhancing the quality of tobacco leaves during aging. According to current research reports, most strains isolated from microorganisms on the surface of flue-cured tobacco during the aging stage are *Bacillus* and *Clostridium* (Mou et al., 2020). *Bacillus* can improve flue gas by secreting amylase, acid protease, pectinase and other hydrolytic enzymes (Chen, 2020) and produce a wide range of volatile flavor compounds through the biodegradation of carotenoids (Hao et al., 2014). Huang et al. (2010) isolated a kind of *Bacillus pumilus* from the surface of tobacco leaves, which can be used for flue-cured tobacco fermentation to improve the aroma, reduce irritation and cover up impurities. Yu et al. (2021) found *Bacillus* in the veins of flue-cured tobacco in Yunnan, Sichuan and Fujian with the ability to produce α- amylase and protease. This study employed 16S rRNA sequencing and the PICRUSt software to investigate the surface bacterial communities and their functional diversity from 14 different grades in Fujian tobacco-producing areas. By analyzing the abundance of the bacterial community on the surface of Fujian flue-cured tobacco, we found that the top 10 dominant bacterial species were *Variovorax*, *Sphingomonas*, *Bacillus*, *Burkholderia-Caballeronia-Paraburkholderia*, *Rhodococcus*, *Methylbacillus*, *Aureimonas Pseudomonas*, *Bacillus* and *Ralstonia*. Among them, in characterizing the distinct microbial communities in light and strong aroma flue-cured tobacco, the main dominant species, *Sphingomonas* sp. Zhang et al. (2020) found to be able to degrade lignin and produce a range of flavor compounds.

The analysis of functional metabolic pathways in the second layer of the KEGG database found that the bacterial community on the surface of flue-cured tobacco played a significant role in carbohydrate metabolism. Studies have shown that *Bacillus amylolyticus*, which secreted complex enzymes (protease, amylase, cellulase, pectinase, and peroxidase), affected the aroma quality and amount of tobacco leaves during the aging process (Qian et al., 2006; Su et al., 2017). The enzyme preparation of *Paenibacillus amylolyticus A17 #,* a target strain screened from the surface of flue-cured tobacco with the ability to degrade starch, was also a complex enzyme system. In addition to producing high levels of amylase, it also produced pectinase and cellulase. In this study, we found that after being treated with the enzyme preparation of *Paenibacillus amyloliquefaciens A17 #*, the content of macromolecular substances in flue-cured tobacco decreased. At the same time, the total sugar and reducing sugar increased. Sugar presents a significant influence on the sensory quality of tobacco, which could weaken the acrimonious taste of the smoke by producing acids and be converted to various aromatic substances by Maillard reaction, caramelization, and pyrolysis reaction (Yang et al., 2014; Yin et al., 2019). Moreover, the sensory evaluation and absorption effect was significantly improved, among which the green gas and woody gas were decreased, the smoke fineness was increased, and the improvement of aroma plumpness was related to the significant reduction of starch, pectin and cellulose content. Meanwhile, increased sugar content in flue-cured tobacco can lead to increased sweetness and better sensory effects.

Our study found that *Bacillus* was associated with plentiful critical aroma components in flue-cured tobacco. *Bacillus subtilis*, *Bacillus coagulans*, *Bacillus circulans*, *Bacillus megaterium*, and *Bacillus thuringiensis* were employed to enhance the development of a desirable aroma and improve the smoking qualities of the tobacco (Kaelin et al., 1994; Zhao et al., 2007). Additionally, the flavor substances of flue-cured tobacco treated with the enzyme preparation of *Paenibacillus amyloliquefaciens A17 #* were significantly increased compared to the control group. Banozic et al. (2020) suggested that starch, a significant metabolite in tobacco, was converted into water-soluble carbohydrates and then into aromatic compounds. During the baking process of tobacco leaves, the Maillard reaction between amino acids formed by protein hydrolysis and the sugar formed by starch hydrolysis is the primary source of an aroma precursor in flue-cured tobacco (Dong et al., 2000).

Furthermore, our study found that *Paenibacillus amyloliquefaciens A17 #* is capable of producing amylase to decompose starch into reducing sugar, which can react with amino acids in the baking process at the end of the enzymatic reaction to produce Maillard products with flavor. After the enzyme treatment, the content of 5-methyl-2-furaldehyde (C17), phenylacetaldehyde (C19), and 2-acetyl pyrrole (C55) increased, of which 5-methyl-2-furaldehyde (C17) has a sweet and burnt smell. Phenylacetaldehyde (C19) has a rose fragrance, and 2-acetyl pyrrole (C55) has a floral fragrance (Li et al., 2017). The significant increase in the content of Maillard reaction products has a positive direct contribution to the improvement of the aroma quality, aroma amount, aftertaste, and total smoking score of flue-cured tobacco. It has an indirect effect on the reduction of impurities and irritation (Shi et al., 2013; Wang et al., 2019). These results indicated that the degradation of starch produces several Maillard reaction products, which could enhance the flavor of tobacco leaves.

In addition, after the enzyme treatment, the content of essential aroma components such as 3-Hydroxy-β-damascone (C13) and benzyl alcohol (C22) increased. Incidentally, phenylacetic acid (C26), which has a sweet and floral fragrance, and dihydroactinidiolide (C32), which has a distinct tobacco aroma, were also increased. 3-Hydroxy-β-damascone (C13), phenylacetic acid (C26), and dihydroactinidiolide (C32) can give flue-cured tobacco a sweet and fruity flavor and finally improve the flavor of flue-cured tobacco (Huang et al., 2010). Recent studies have shown that carotenoids, as precursors that can produce many important aroma components, play an essential role in the formation of the aroma quality of tobacco (Xu et al., 2012). The degradation of carotenoids can produce 3-Hydroxy-β-damascone (C13) with a sweet and floral fragrance, making tobacco aroma mellow and delicate. Peroxidase, which was the main enzyme (Zelena et al., 2009) that could degrade carotenoids and produce fragrance, acted on C9-C10/C9’-C10’and C7-C8/C7’-C8’ double bond positions to produce β-ionone (C10), dihydroactinidiolide (C32), and other aroma substances (Zorn et al., 2003). Incidentally, Rodriguez-Bustamante et al. found that the mixed fermentation of *Astoria* and *Bacillus amyloides* can catalyze the degradation of xanthoxanthin and produce aroma components (Rodriguez-Bustamante et al., 2005). Therefore, in this study, there may be a potential relationship between the production of these aroma substances and the carotenoids degradation of tobacco leaves by *Paenibacillus amylolyticus A17 #*.

## 5. Conclusion

This study analyzed the diversity of the bacterial community on the surface of Fujian flue-cured tobacco and the flavor of different grades of tobacco. The results revealed that *Bacillus* was the dominant bacteria in the abundance of the bacterial community on the surface of Fujian flue-cured tobacco, and it was positively correlated with many volatile components and sweet flavor in tobacco leaves. The target strain of *Paenibacillus amylolyticus A17 #*, which can produce amylase on the surface of flue-cured tobacco leaves, was further screened out. The enzyme preparation of *Paenibacillus amylolyticus A17 #* can reduce the content of starch, pectin, and cellulose in flue-cured tobacco after treatment, and the sensory indicators such as aroma quantity, sweetness, aroma quality, strength, concentration, and aftertaste are significantly improved. When the enzyme preparation of *Paenibacillus amylolyticus A17 #* acts on flue-cured tobacco, leading to a significant rise in the concentration of sweet and fruity flavor compounds such as phenylacetic acid, dihydroactinidiolide and ethyl palmitate. The results presented here serve as a crucial theoretical guide for enhancing the taste of flue-cured tobacco using microbial enzyme preparations.

## Data availability statement

The datasets presented in this study can be found in online repositories. The sequencing data and accession number(s) can be found below: https://www.ncbi.nlm.nih.gov/sra/PRJNA962708.

## Author contributions

YG: conceptualization, methodology, validation, investigation, formal analysis, writing–original draft, and visualization. JL: methodology, validation, writing–review and editing, and supervision. XD, YC, and SC: methodology and investigation. HH: visualization and investigation. LN: validation and investigation. TL, WH, and JZ: formal analysis and data curation. ZJ: formal analysis and validation. JF and WZ: methodology, validation, writing–review and editing, supervision, and project administration. All authors contributed to the article and approved the submitted version.

## Funding

This work was supported by the Science and Technology Project of CNTC (Grant/Award number: 110202202022). The authors declare that this study received funding from Science and Technology Project of China Tobacco Fujian Industrial Co., Ltd. China (Grant/Award number: FJZYKJJH2020008). The funder was not involved in the study design, collection, analysis, interpretation of data, the writing of this article, or the decision to submit it for publication.

## Conflict of interest

JL, XD, YC, SC, TL, WH, JZ, ZJ, and JF were employed by Technology Center, China Tobacco Fujian Industrial Co., Ltd.

The remaining authors declare that the research was conducted in the absence of any commercial or financial relationships that could be construed as a potential conflict of interest.

## Publisher’s note

All claims expressed in this article are solely those of the authors and do not necessarily represent those of their affiliated organizations, or those of the publisher, the editors and the reviewers. Any product that may be evaluated in this article, or claim that may be made by its manufacturer, is not guaranteed or endorsed by the publisher.

## Footnotes

^1^https://www.arb-silva.de/
