# Supplementary material for: Application of starch degrading bacteria from tobacco leaves in improving the flavor of flue-cured tobacco
Source: Front Microbiol. 2023 Jun 27;14:1211936. doi: 10.3389/fmicb.2023.1211936 (PMC10335769; doi:10.3389/fmicb.2023.1211936)
Supplement: Supplementary file 1 [file Table_1.pdf]

Appenndix Tab. 1 Changes of volatile substances in tobacco leaves before and after enzyme treatment

NO.	Volatile components	NO.	Volatile components
C1	2,6,6-trimethylcyclohex-2-ene-1,4-dione	C35	Methyl 9-methyltetradecanoate
C2	2,3-dihydro-3,5-dihydroxy-6-methyl-4H-Pyran-4-one	C36	Citronellyl acetate
C3	6-Propyl-5,6-dihydro-2H-pyran-2-one	C37	Ethyl 13-methyltetradecanoate
C4	2-Hydroxymethyl-5-hydroxy-g-pyrone	C38	Methyl 8,11,14,17-eicosatetraenoate
C5	Solanone	C39	Methyl palmitate
C6	β-Damascenone	C40	Ethyl 6,9,12-hexadecatrienoate
C7	Damascone	C41	Ethyl palmitate
C8	Geranylacetone	C42	Methyl 14-methylhexadecanoate
C9	4-(2,6,6-Trimethylcyclohexa-1,3-dienyl)but-3-en-2-one	C43	Methyl linoleate
C10	beta-Ionone	C44	Methyl Linolenate
C11	MegastigmatrienoneA	C45	Phytol
C12	MegastigmatrienoneB	C46	Methyl stearate
C13	3-Hydroxy-*β*-damascone	C47	Ethyl linoleate
C14	MegastigmatrienoneC	C48	Ethyl linolenic acid
C15	MegastigmatrienoneD	C49	Ethyl stearate
C16	Furfural	C50	L-(-)-Nicotine
C17	5-Methyl-2-furaldehyde	C51	α-Nicotine
C18	Phenylacetaldehyde	C52	Myosmine
C19	Benzeneacetaldehyde	C53	Nicotyrine
C20	2-Phenylcrotonaldehyde	C54	2,3-Dimethylmaleic anhydride
C21	Vanillin	C55	2-acetyl pyrrole
C22	Benzyl alcohol	C56	2-ethyl-3-methylmaleimide
C23	Phenylethyl Alcohol	C57	1,4,6-trimethyltetralin
C24	3-Oxo-*α*-ionol	C58	2,3,6-Trimethylnaphthoquinone
C25	Benzoic acid	C59	3-(4,8,12-Trimethyltridecyl) furan
C26	Phenylacetic acid	C60	3,5,5-trimethyl-Cyclohexene
C27	Tetradecanoic acid	C61	1,5,8-*p*-Menthatriene
C28	n-Hexadecanoic acid	C62	Cembrene
C29	Linoleic acid	C63	Neophytadiene
C30	*α*-Linolenic acid	C64	Octadecane
C31	Methyl phenylacetate	C65	Tridecane
C32	Dihydroactinidiolide	C66	(6E,10E)-7,11,15-Trimethyl-3-methylene-1,6,10,14-hexadecatetraene
C33	Methyl myristate	C67	6,6-dimethyl-3-methylene-Bicyclo[3.1.1]heptane
C34	Ethyl myristate	C68	3-Tetradecene
